# Comparative Adverse Event Reporting of Asparaginase-Containing Induction Regimens in Pediatric Acute Lymphoblastic Leukemia Using the United States Food and Drug Administration Adverse Event Reporting System

**DOI:** 10.7759/cureus.108119

**Published:** 2026-05-01

**Authors:** Toru Ogura, Chihiro Shiraishi

**Affiliations:** 1 Clinical Research Support Center, Mie University Hospital, Tsu, JPN; 2 Department of Pharmaceutical Sciences for Health Crisis Management, Faculty of Pharmaceutical Sciences, Fukuoka University, Fukuoka, JPN

**Keywords:** acute lymphoblastic leukemia, adverse event reporting system, asparaginase, induction therapy, pediatric oncology, pharmacovigilance

## Abstract

Background

Asparaginase (ASP) is a key component of pediatric acute lymphoblastic leukemia (ALL) therapy, but its use is limited by concerns about hypersensitivity, hepatic dysfunction, pancreatitis, thrombosis, and other toxicities. Comparative information on adverse event (AE) reporting signals for ASP-containing versus ASP-free induction regimens differing only by ASP inclusion remains limited in real-world pharmacovigilance data.

Objectives

To compare AE reporting patterns between ASP-containing and ASP-free regimen pairs in pediatric ALL and to characterize regimen-specific reporting signals across major AE categories.

Methods

Pediatric ALL reports submitted to the United States Food and Drug Administration Adverse Event Reporting System (FAERS) from 2004 to 2025 were identified using ALL indication terms. As FAERS does not reliably encode treatment phase, we operationally restricted the analysis to induction-oriented reports by requiring both a corticosteroid and vincristine as the backbone regimen, limiting concomitant drugs to a predefined set of induction agents, and excluding reports containing drugs typically used in consolidation, maintenance, targeted therapy, or immunotherapy. Comparisons were restricted to paired regimens in which ASP inclusion was the defining difference within the same induction-oriented backbone. AEs were grouped into 10 predefined categories. Reporting odds ratios (RORs), adjusted RORs (aRORs), and, when necessary, modified RORs were estimated using logistic regression, adjusting for age, sex, and continent when feasible. Findings were interpreted as spontaneous reporting signals rather than incidence estimates, and greater emphasis was placed on AE categories for which both the unadjusted ROR and adjusted ROR showed concordant statistically significant associations.

Results

Among pediatric ALL reports meeting the induction-oriented criteria, five regimen pairs met the predefined case-count threshold for comparative analysis, comprising 740 reports in total. AE reporting patterns differed across regimen pairs and AE categories, reflecting regimen‑specific disproportionality in FAERS data. Increased reporting signals were observed for infections, nervous system disorders, pancreatitis, and gastrointestinal disorders in selected ASP-containing regimens, whereas lower reporting signals were observed for hepatobiliary and hematologic disorders in other regimen pairs. Overall, the direction and magnitude of the signals differed across AE categories and induction backbones.

Conclusions

In pediatric ALL induction therapy, ASP-containing regimens showed regimen-specific AE reporting signals in FAERS rather than evidence of true incidence differences or causal effects. These patterns may reflect the influence of accompanying chemotherapy components as well as reporting bias, including notoriety bias, whereby well-recognized ASP toxicities may be more likely to be attributed and reported. As FAERS is a spontaneous reporting system, these findings are hypothesis-generating and support prospective studies to clarify the safety profile of ASP across distinct induction regimens.

## Introduction

Acute lymphoblastic leukemia (ALL) is the most common childhood malignancy, accounting for the majority of pediatric leukemias. The estimated global incidence is approximately two to three cases per 100,000 children per year, corresponding to more than 40,000 new cases annually worldwide [1,2]. Despite substantial therapeutic advances that have raised five‑year survival rates above 80-90% in many high‑income countries and markedly reduced ALL‑related mortality, the disease remains a leading cause of cancer‑related death among children globally [1,2]. Contemporary pediatric ALL treatment relies on multi‑agent chemotherapy delivered in sequential phases (induction, consolidation, and maintenance), with additional risk‑adapted intensification and, in selected cases, targeted or immunotherapeutic approaches [3]. Among these phases, remission induction is critical for rapidly debulking leukemic blasts and achieving complete remission, which is essential for long-term disease control [4]. Pediatric induction regimens typically combine glucocorticoids and vincristine (VCR) with other agents such as asparaginase (ASP), anthracyclines (ANTR, including doxorubicin), alkylating agents (ALKY, including cyclophosphamide), and antimetabolites (AMT, including methotrexate), with various combinations adopted across nations and cooperative study groups [5,6].

ASP is a key component of many pediatric‑inspired induction regimens because it depletes circulating asparagine, thereby impairing leukemic cell survival and contributing to high rates of minimal residual disease negativity and improved cure outcomes [7-9]. However, its use is limited by clinically important adverse events (AEs), including hypersensitivity, hepatic dysfunction, pancreatitis, thrombosis, and metabolic disturbances, often leading to dose reduction or discontinuation. In clinical practice, ASP or other induction agents may be reduced or omitted in response to toxicity, resulting in real‑world variations in induction regimens that differ in ASP inclusion and accompanying agents. Previous clinical trials and cohort studies have reported ASP‑related toxicities; however, many of these investigations feature heterogeneous regimens, include subsequent treatment phases (e.g., consolidation or maintenance), or focus on selected high‑risk subgroups [8,10,11]. Consequently, there is limited systematic evidence comparing induction regimens that share a common base regimen but differ specifically by ASP use, particularly under real‑world, multinational conditions.

Each induction agent has distinct mechanisms of action and pharmacokinetic properties, and interactions among coadministered drugs may contribute to differences in observed reporting patterns across specific multi-agent combinations [7]. Therefore, isolating regimen pairs that differ only in the inclusion of ASP provides a pragmatic way to explore how ASP may alter AE patterns in clinical settings. Since it is impractical to conduct prospective clinical trials covering all possible induction drug combinations, systematic comparative data on ASP-containing versus ASP-free regimens are scarce, especially in analyses isolating the induction phase. Large post‑marketing AE reporting databases that capture reports from multiple countries therefore provide an opportunity to explore regimen-specific AE reporting patterns under clinical conditions, while acknowledging the inherent limitations of spontaneous reporting data. However, few studies have directly compared ASP-containing and ASP-free induction regimens that are otherwise matched within the same treatment backbone, particularly using multinational real-world pharmacovigilance data.

This study aimed to investigate AE reporting signals associated with ASP in steroid (STR, including prednisone, prednisolone, methylprednisolone, and dexamethasone) and VCR-based induction regimens for pediatric ALL using the United States Food and Drug Administration Adverse Event Reporting System (FAERS) [12]. Specifically, we compared patient characteristics, AE patterns, and disproportionality signals between ASP‑containing and ASP‑free regimens to evaluate potential differences in AE reporting patterns.

## Materials and methods

Data source

This study was based on safety reports submitted to the FAERS, a spontaneous reporting database for post‑marketing pharmacovigilance. FAERS contains case-level information on patient demographics, reported drugs, indications, and AEs, and is released as quarterly data files. As a spontaneous reporting system, FAERS was subject to underreporting, reporting bias, duplicate submissions, and missing or incomplete information. For this analysis, all available quarterly datasets from the first quarter of 2004 (2004Q1) through 2025Q4 were downloaded from the official FAERS website on January 31, 2026. Data from 2004Q1 to 2012Q3 were obtained in the legacy Adverse Event Reporting System (AERS) format (file names: aers_ascii_yyyyQq.zip, where “yyyy” denotes the year and “q” the quarter). From 2012Q4 onward, data were obtained in the FAERS format (faers_ascii_yyyyQq.zip). Each quarterly archive includes core ASCII (American Standard Code for Information Interchange) text tables, DEMOyyQq.txt (patient demographics and administrative information), DRUGyyQq.txt (drug exposure), REACyyQq.txt (AEs), INDIyyQq.txt (indications), and THERyyQq.txt (therapy start and end dates), where “yy” represents the final two digits of the year. The AERS and FAERS files were merged into a single, harmonized dataset following the official FAERS documentation. In FAERS, when follow-up information is submitted for a previously reported case, the original report is not overwritten; instead, a new case version is created with the same {caseid} and an incremented {caseversion} value. Therefore, when multiple versions of the same {caseid} were present, only the most recent entry, identified by the highest {caseversion} value, was retained. For older AERS data lacking explicit versioning, follow-up submissions were resolved using {ISR} and {CASE}, with the chronologically latest record preserved. Variable names in the original FAERS/AERS files are shown in curly brackets for clarity and reproducibility. Data cleaning and standardization were performed before analysis. Key patient variables, including sex, age, body weight, and reporter country or region, were reviewed and normalized. Reporter countries were further classified into continental regions (North America, Latin America, Asia, Europe, Oceania, and Africa). Age values were uniformly converted to years and body weight to kilograms. Formatting inconsistencies in the raw text files were corrected solely to restore the intended tabular structure and line breaks, specifically, missing line breaks in DRUG11Q2.txt (line 322,967), DRUG11Q3.txt (line 247,896), and DRUG11Q4.txt (line 446,738), without altering any clinical content or variable values.

All data used in this study were fully de‑identified and obtained from a publicly accessible regulatory database. No patient contact or identifiable data were involved, and, therefore, ethics approval was not required.

Study design

This retrospective pharmacovigilance study evaluated disproportionality signals in AE reporting among children with ALL receiving STR‑ and VCR‑based induction therapy, with a specific focus on the presence or absence of ASP. The study was reported in accordance with the RECORD (Reporting of studies Conducted using Observational Routinely-collected health Data) statement [13] and informed by the principles of the International Society for Pharmacoepidemiology Good Pharmacoepidemiology Practices [14], where applicable. Induction regimens were categorized according to the specific combination of coadministered agents, excluding ASP, which defined the base regimen. Each base regimen was then divided into two subgroups according to the absence or presence of ASP (ASP- or ASP+), and comparisons were performed between the ASP- and ASP+ groups within each base regimen, enabling a more direct and clinically intuitive assessment of ASP-associated reporting patterns. To ensure stable estimation of disproportionality signals, the analysis was restricted to regimen pairs in which both the ASP-negative and ASP-positive groups contained ≥40 cases. Pediatric ALL cases were defined as individuals aged 0-14 years whose indication term in the {indi_pt} field corresponded to ALL. We defined pediatric ALL as age 0-14 years to focus on a more homogeneous pediatric cohort, because treatment settings and regimen selection may become more heterogeneous in older adolescents [15,16]. A detailed list of included indication terms is provided in Appendix A. Induction regimens included six agents: ASP, STR, VCR, ANTR, ALKY, and AMT. This study specifically focused on regimens that always contained both STR and VCR. When the {prod_ai} variable (product active ingredient) was available, these drugs were identified by their generic names. For periods when {prod_ai} was not provided, generic and brand names listed in the {drugname} field were used. Regimen assignment was based on the {role_cod} variable (code for drug's reported role in event) for each drug record in FAERS. Only drugs coded as primary suspect (PS) or secondary suspect (SS) were used to define study regimens; concomitant and interacting drugs were excluded from regimen assignment. For each regimen, at least one drug had to be coded as PS, with the remaining regimen-defining drugs coded as SS. The complete list of generic and brand names for each drug is summarized in Appendix B. To restrict the analysis to induction therapy, reports were included only when all recorded concomitant drugs belonged to the predefined induction components. Cases were excluded if they involved any agents typically administered outside the remission-induction phase, including pyrimidine analogs (PYR, including cytarabine), topoisomerase inhibitors (TOPO, including etoposide), purine analogs (PNA, including mercaptopurine), tyrosine kinase inhibitors (TKI, including imatinib and dasatinib), bispecific T-cell engager antibodies (BiTE, including blinatumomab), or anti-CD20 antibodies (CD20, including rituximab), as listed in Appendix B. Reports were also excluded when the recorded drug therapy start date {start_dt} occurred exclusively after the AE date {event_dt}. For cases with multiple therapy intervals, a report was retained if at least one treatment interval began on or before the AE date. As described in the *Data source* section, after retaining only the most recent version for each {caseid}, any remaining potential duplicate reports, defined as records sharing age, sex, weight, reporter country, event date, preferred terms, and drug combinations, were manually reviewed and collapsed into a single case. During data cleaning, weights of ≥120 kg in the 0-14-year cohort were treated as implausible and excluded if the discrepancy could not be resolved by checking age and other available clinical information.

AEs were grouped into predefined clinical categories: infections, nervous system disorders, hematologic disorders, hepatobiliary disorders, pancreatitis, gastrointestinal disorders, thrombotic disorders, metabolic and endocrine disorders, hypersensitivity, and respiratory disorders. These categories were selected a priori to capture the major clinically recognized toxicities of ASP and the principal organ-system outcomes relevant to pediatric ALL induction therapy. Pancreatitis and thrombotic disorders were retained as separate categories because of their distinct clinical importance and management implications. AEs were coded using Preferred Terms from the Medical Dictionary for Regulatory Activities (MedDRA), and detailed lists of the terms assigned to each category are provided in Appendix C. Each quarterly FAERS dataset used the MedDRA version included in the original release of that dataset, which reflects the MedDRA version current at the time of release; no additional recoding or harmonization was performed after release, and the specific versions are listed in Appendix D. AE categories were evaluated at the report level, and a single case report could contribute to more than one category if AEs from multiple categories were present. Within each category, each case was counted only once, even if multiple preferred terms were reported.

Statistical analyses

Continuous variables were summarized as medians with first and third quartiles. Categorical variables were described as counts with reporting proportions (RPs) [17]. RP was calculated as (number of pediatric ALL cases with the characteristic of interest) / (total number of pediatric ALL cases reported in FAERS). Unknown categories were explicitly reported as separate levels and were not imputed. For each induction regimen group, baseline characteristics (sex, age, weight, and continent) were first summarized, followed by the reporting frequencies of AE categories. To evaluate differences in AE reporting between ASP-containing and ASP-free regimens, we calculated reporting odds ratios (RORs) [18] with 95% confidence intervals (CIs) for each paired comparison in which the only difference was the ASP component. RORs were obtained from univariate logistic regression models, treating the presence of a given AE category as the dependent variable and regimen status (with vs. without ASP) as the independent variable. To account for potential confounding by demographic and regional factors, we estimated adjusted reporting odds ratios (aRORs) using multivariable logistic regression models, including age, sex, and reporter region as covariates. Because weight was frequently missing and missingness in FAERS could not be assumed to be random, weight was not included in the multivariable models to avoid excessive case loss and unstable estimates. As a result, residual confounding related to body size and weight-dependent dosing may remain in the adjusted analyses. Given the limited covariate information available in FAERS, the remaining missing values were handled by complete-case analysis for the covariates included in each model, and no formal imputation procedures were applied. The {reporter_country} variable was recoded into continent-level categories, and only North America, Asia, and Europe were included in regression models because reports from Latin America, Oceania, and Africa were very limited. North America was used as the reference category for continent, and female was used as the reference for sex. Because the regimen variable was included in all models, only the covariate set was varied across candidate models, and the model with the lowest Akaike information criterion [19] was selected as the final multivariable model for each analysis. For each regimen pair, ASP-containing regimens were compared with their corresponding ASP-free reference regimens to reduce confounding related to differences in the underlying induction base regimen. Statistical significance was assessed using two-sided tests, and statistical significance was defined as p < 0.05. In interpreting the results, greater emphasis was placed on AE categories for which both the unadjusted ROR and adjusted aROR showed concordant directions of association and statistically significant estimates (p < 0.05), thereby mitigating the impact of missing data on aROR estimates by requiring corroboration from the unadjusted analysis, which included all available cases. All statistical analyses were conducted using R software version 4.4.1 (R Foundation for Statistical Computing, Vienna, Austria). For the primary analysis, regimen pairs were retained only when both the ASP‑negative and ASP‑positive groups contained ≥40 cases.

As sensitivity analyses, the following approaches were performed: (1) continent-specific subgroup analyses (Europe, North America, Asia); (2) inclusion of regimen pairs with 10 to 39 cases in at least one group; (3) analyses permitting prohibited concomitant drugs; and (4) extension of age definition to 0-17 years.

## Results

Patient background

**Figure 1:**
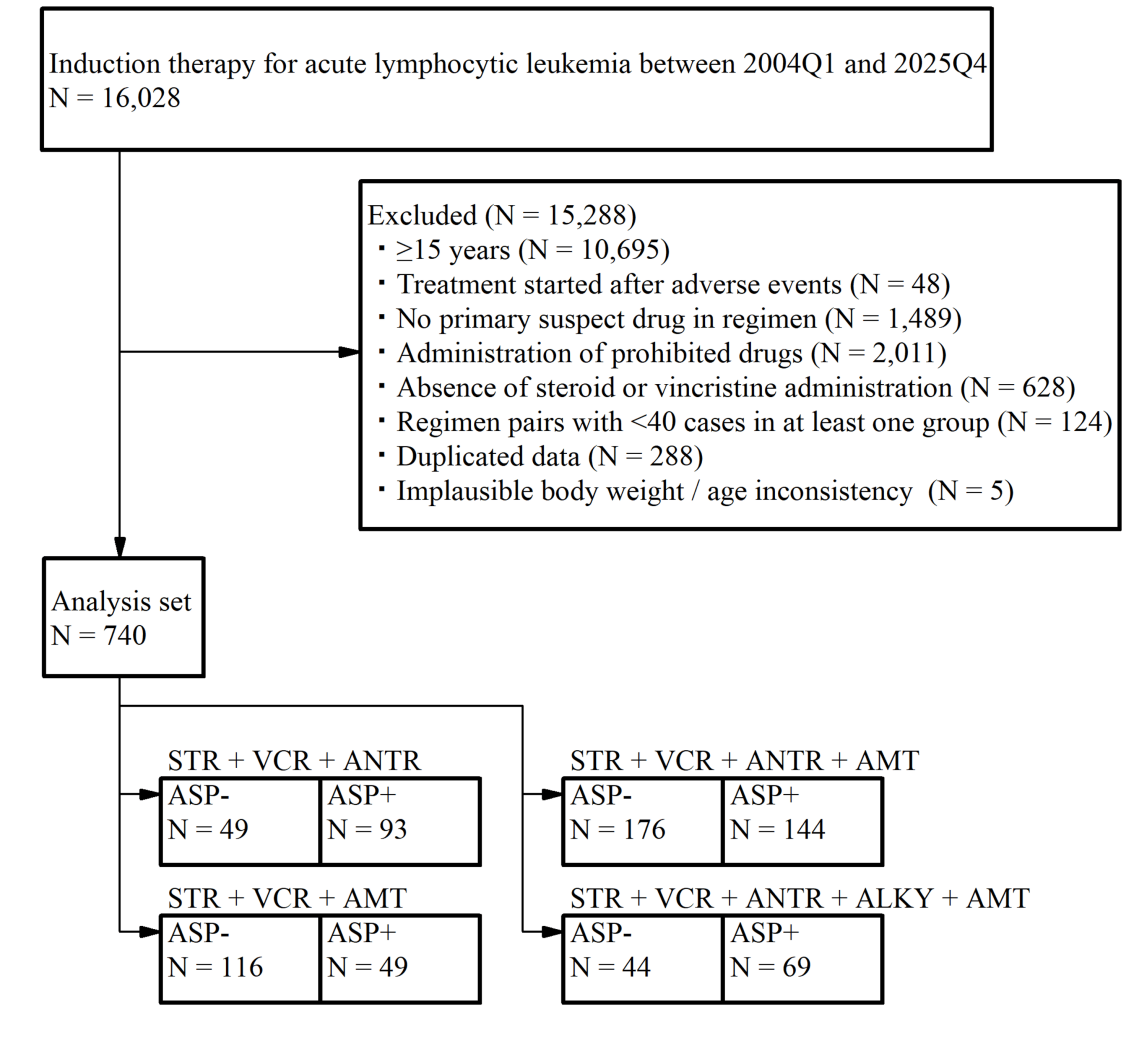
Flowchart of case selection and induction regimen classification in pediatric acute lymphoblastic leukemia. ALKY, alkylating agent; AMT, antimetabolite; ANTR, anthracyclines; ASP-, asparaginase not added to base regimen; ASP+, asparaginase added to base regimen; STR, steroid; VCR, vincristine.

From the FAERS database (2004Q1-2025Q4), 740 pediatric ALL cases met the predefined criteria for ALL and induction‑phase treatment with STR‑ and VCR‑based regimens. Across these cases, five base regimen combinations were identified, each of which included ≥40 reports in both the ASP- and ASP+ groups (STR + VCR + ANTR; STR + VCR + AMT; STR + VCR + ANTR + AMT; and STR + VCR + ANTR + ALKY + AMT). These regimen combinations are presented in parallel in the study flowchart (Figure 1). In addition to the five regimen pairs included in the primary analysis, several regimen pairs were excluded because at least one group had <40 cases or because prohibited concomitant drugs were present. These excluded pairs are summarized in Appendix E.

As shown in Table 1, sex distribution varied by regimen, but males generally accounted for a slightly larger proportion of cases in most groups. The degree of between-group sex imbalance also varied across regimen pairs, with some comparisons showing more pronounced differences than others. Median age at the time of reporting was consistently in early to mid‑childhood across all regimen groups. Body weight data were frequently missing in all groups. Among reports with available values, median weights were broadly compatible with the observed age distributions, but interpretation remains limited by the high proportion of unknown entries. Reports originated from multiple continents, underscoring the international nature of the FAERS dataset. The distribution of reporter continent differed across regimen groups and should be interpreted descriptively rather than as evidence of treatment preference. In this cohort, ASP+ cases were relatively more common from Asia in several regimens, whereas ASP- cases were more often reported from North America and Europe in some groups. Overall, reports from Latin America, Oceania, and Africa were uncommon.

**Table 1:** Baseline characteristics of pediatric acute lymphoblastic leukemia cases across induction regimen groups. Sex and country are summarized as frequencies (reporting proportions). Age and weight are summarized as medians and first and third quartiles. Unknown for each variable is summarized as frequency (reporting proportion). ALKY, alkylating agent; AMT, antimetabolite; ANTR, anthracyclines; ASP-, asparaginase not added to base regimen; ASP+, asparaginase added to base regimen; Q1, first quartile; Q3, third quartile; STR, steroid; VCR, vincristine.

Base regimen	STR + VCR + ANTR		STR + VCR + AMT		STR + VCR + ANTR + AMT		STR + VCR + ANTR + ALKY + AMT	
	ASP-	ASP+	ASP-	ASP+	ASP-	ASP+	ASP-	ASP+
	N = 49	N = 93	N = 116	N = 49	N = 176	N = 144	N = 44	N = 69
Sex, n (%)								
Female	21 (42.9)	40 (43.0)	28 (24.1)	23 (46.9)	91 (51.7)	53 (36.8)	5 (11.4)	27 (39.1)
Male	28 (57.1)	51 (54.8)	77 (66.4)	25 (51.0)	79 (44.9)	90 (62.5)	38 (86.4)	41 (59.4)
Unknown	0 (0.0)	2 (2.2)	11 (9.5)	1 (2.0)	6 (3.4)	1 (0.7)	1 (2.3)	1 (1.4)
Age, years								
Median	5.0	4.0	5.0	8.0	8.0	8.0	7.0	7.0
Q1-Q3	3.0-11.0	2.0-8.0	3.0-8.0	5.0-10.0	3.8-11.0	5.0-10.0	5.0-11.0	4.0-9.0
Unknown, n (%)	0 (0.0)	0 (0.0)	0 (0.0)	0 (0.0)	0 (0.0)	0 (0.0)	0 (0.0)	0 (0.0)
Weight, kg								
Median	18.6	16.8	22.5	22.0	37.0	30.0	29.9	18.0
Q1-Q3	12.7-35.2	13.5-51.0	19.0-27.0	13.5-24.4	20.7-43.5	20.0-35.4	18.5-31.6	14.3-44.8
Unknown, n (%)	37 (75.5)	80 (86.0)	94 (81.0)	33 (67.3)	141 (80.1)	129 (89.6)	33 (75.0)	62 (89.9)
Continent, n (%)								
Europe	41 (83.7)	33 (35.5)	63 (54.3)	8 (16.3)	113 (64.2)	44 (30.6)	25 (56.8)	10 (14.5)
North America	5 (10.2)	8 (8.6)	33 (28.4)	15 (30.6)	44 (25.0)	32 (22.2)	14 (31.8)	5 (7.2)
Asia	1 (2.0)	48 (51.6)	17 (14.7)	17 (34.7)	16 (9.1)	48 (33.3)	3 (6.8)	45 (65.2)
Latin America	1 (2.0)	0 (0.0)	0 (0.0)	0 (0.0)	0 (0.0)	2 (1.4)	1 (2.3)	0 (0.0)
Africa	0 (0.0)	0 (0.0)	0 (0.0)	0 (0.0)	1 (0.6)	3 (2.1)	0 (0.0)	0 (0.0)
Oceania	0 (0.0)	1 (1.1)	1 (0.9)	0 (0.0)	0 (0.0)	1 (0.7)	0 (0.0)	0 (0.0)
Unknown	1 (2.0)	3 (3.2)	2 (1.7)	9 (18.4)	2 (1.1)	14 (9.7)	1 (2.3)	9 (13.0)

Adverse events

Table 2 summarizes the distribution of AE categories across regimen groups, focusing on paired regimens that differed only in the inclusion or exclusion of ASP.

**Table 2:** Reporting frequencies of adverse event categories by induction regimen and asparaginase use. Data are summarized as frequencies (reporting proportions). ALKY, alkylating agent; AMT, antimetabolite; ANTR, anthracyclines; ASP-, asparaginase not added to base regimen; ASP+, asparaginase added to base regimen; STR, steroid; VCR, vincristine.

Base regimen	STR + VCR + ANTR		STR + VCR + AMT		STR + VCR + ANTR + AMT		STR + VCR + ANTR + ALKY + AMT	
	ASP-	ASP+	ASP-	ASP+	ASP-	ASP+	ASP-	ASP+
	N = 49	N = 93	N = 116	N = 49	N = 176	N = 144	N = 44	N = 69
Infections	8 (16.3)	24 (25.8)	26 (22.4)	11 (22.4)	29 (16.5)	39 (27.1)	4 (9.1)	20 (29.0)
Nervous system disorders	3 (6.1)	20 (21.5)	11 (9.5)	14 (28.6)	19 (10.8)	35 (24.3)	6 (13.6)	20 (29.0)
Hematologic disorders	20 (40.8)	20 (21.5)	26 (22.4)	13 (26.5)	46 (26.1)	47 (32.6)	21 (47.7)	17 (24.6)
Hepatobiliary disorders	6 (12.2)	11 (11.8)	23 (19.8)	4 (8.2)	28 (15.9)	6 (4.2)	5 (11.4)	5 (7.2)
Pancreatitis	3 (6.1)	4 (4.3)	5 (4.3)	0 (0.0)	5 (2.8)	18 (12.5)	4 (9.1)	1 (1.4)
Gastrointestinal disorders	11 (22.4)	9 (9.7)	11 (9.5)	4 (8.2)	33 (18.8)	49 (34.0)	4 (9.1)	11 (15.9)
Thrombotic disorders	1 (2.0)	14 (15.1)	20 (17.2)	8 (16.3)	26 (14.8)	9 (6.2)	6 (13.6)	12 (17.4)
Metabolic and endocrine disorders	5 (10.2)	8 (8.6)	8 (6.9)	5 (10.2)	22 (12.5)	28 (19.4)	6 (13.6)	9 (13.0)
Hypersensitivity	0 (0.0)	1 (1.1)	0 (0.0)	0 (0.0)	2 (1.1)	1 (0.7)	0 (0.0)	0 (0.0)
Respiratory disorders	0 (0.0)	2 (2.2)	4 (3.4)	6 (12.2)	7 (4.0)	5 (3.5)	2 (4.5)	4 (5.8)

Table 3 presents only AE categories with concordant statistically significant ROR and aROR results.

**Table 3:** The ROR and aROR of adverse event categories. N indicates the number of reports included in each model. Only adverse event categories with both ROR and aROR showing statistically significant associations were included. Multivariate models were selected based on the lowest Akaike information criterion among candidate models. ALKY, alkylating agent; AMT, antimetabolite; ANTR, anthracycline; aROR, adjusted reporting odds ratio; ASP+, asparaginase added to base regimen; CI, confidence interval; ROR, reporting odds ratio; STR, steroid; VCR, vincristine; -, not selected in multivariate analysis.

Adverse event category	Variable	Reference	Comparison	Univariate analysis			Multivariate analysis		
				N	ROR (95% CI)	p-value	N	aROR (95% CI)	p-value
Infections	Regimen	STR + VCR + ANTR + AMT	ASP+	320	1.883 (1.095−3.237)	0.022	290	1.967 (1.028−3.762)	0.041
	Sex	Female	Male	313	1.550 (0.882−2.725)	0.127		1.572 (0.850−2.906)	0.150
	Age	Per 1-year increase		320	0.917 (0.854−0.984)	0.016		0.911 (0.843−0.985)	0.020
	Continent	Europe	North America	297	1.760 (0.898−3.448)	0.099		1.786 (0.888−3.594)	0.104
	Continent	Europe	Asia		2.066 (1.034−4.130)	0.040		1.259 (0.558−2.843)	0.579
Nervous system disorders	Regimen	STR + VCR + AMT	ASP+	165	3.818 (1.588−9.181)	0.003	142	4.464 (1.615−12.342)	0.004
	Sex	Female	Male	153	0.707 (0.293−1.708)	0.441		0.926 (0.352−2.440)	0.877
	Age	Per 1-year increase		165	1.080 (0.959−1.217)	0.202		-	-
	Continent	Europe	North America	153	1.813 (0.676−4.864)	0.237		1.533 (0.513−4.586)	0.445
	Continent	Europe	Asia		1.476 (0.479−4.548)	0.498		0.738 (0.208−2.613)	0.637
	Regimen	STR + VCR + ANTR + AMT	ASP+	320	2.653 (1.442−4.881)	0.002	313	2.616 (1.397−4.898)	0.003
	Sex	Female	Male	313	0.863 (0.478−1.559)	0.625		0.747 (0.404−1.380)	0.351
	Age	Per 1-year increase		320	1.045 (0.969−1.128)	0.254		1.051 (0.971−1.138)	0.217
	Continent	Europe	North America	297	1.860 (0.863−4.008)	0.113		-	-
	Continent	Europe	Asia		3.223 (1.535−6.767)	0.002		-	-
Hematologic disorders	Regimen	STR + VCR + ANTR + ALKY + AMT	ASP+	113	0.358 (0.160−0.802)	0.013	111	0.393 (0.168−0.922)	0.032
	Sex	Female	Male	111	2.307 (0.890−5.976)	0.085		1.761 (0.644−4.821)	0.271
	Age	Per 1-year increase		113	0.995 (0.893−1.109)	0.924		0.973 (0.867−1.091)	0.635
	Continent	Europe	North America	102	2.901 (0.912−9.228)	0.071		-	-
	Continent	Europe	Asia		0.242 (0.081−0.724)	0.011		-	-
Hepatobiliary disorders	Regimen	STR + VCR + ANTR + AMT	ASP+	320	0.230 (0.092−0.572)	0.002	320	0.230 (0.092−0.572)	0.002
	Sex	Female	Male	313	0.732 (0.359−1.494)	0.391		-	-
	Age	Per 1-year increase		320	1.011 (0.923−1.108)	0.806		-	-
	Continent	Europe	North America	297	0.355 (0.131−0.964)	0.042		-	-
	Continent	Europe	Asia		0.163 (0.037−0.707)	0.015		-	-
Pancreatitis	Regimen	STR + VCR + ANTR + AMT	ASP+	320	4.886 (1.767−13.511)	0.002	290	6.576 (2.208−19.590)	0.001
	Sex	Female	Male	313	0.766 (0.327−1.792)	0.538		0.575 (0.219−1.511)	0.262
	Age	Per 1-year increase		320	1.135 (1.010−1.276)	0.034		-	-
	Continent	Europe	North America	297	5.193 (1.890−14.273)	0.001		4.268 (1.499−12.156)	0.007
	Continent	Europe	Asia		0.812 (0.159−4.133)	0.802		0.425 (0.080−2.264)	0.316
Gastrointestinal disorders	Regimen	STR + VCR + ANTR	ASP+	142	0.370 (0.142−0.967)	0.043	140	0.373 (0.141−0.990)	0.048
	Sex	Female	Male	140	2.625 (0.897−7.682)	0.078		2.669 (0.899−7.921)	0.077
	Age	Per 1-year increase		142	0.976 (0.866−1.100)	0.692		-	-
	Continent	Europe	North America	136	0.357 (0.043−2.979)	0.341		-	-
	Continent	Europe	Asia		0.280 (0.076−1.030)	0.056		-	-
	Regimen	STR + VCR + ANTR + AMT	ASP+	320	2.235 (1.340−3.729)	0.002	290	2.544 (1.402−4.616)	0.002
	Sex	Female	Male	313	0.805 (0.486−1.333)	0.399		0.792 (0.458−1.369)	0.403
	Age	Per 1-year increase		320	0.960 (0.900−1.024)	0.215		0.973 (0.907−1.044)	0.445
	Continent	Europe	North America	297	0.707 (0.368−1.359)	0.298		0.618 (0.313−1.221)	0.166
	Continent	Europe	Asia		0.884 (0.454−1.720)	0.716		0.659 (0.310−1.399)	0.278
Thrombotic disorders	Regimen	STR + VCR + ANTR + AMT	ASP+	320	0.385 (0.174−0.850)	0.018	297	0.383 (0.149−0.982)	0.046
	Sex	Female	Male	313	0.962 (0.462−2.002)	0.917		-	-
	Age	Per 1-year increase		320	1.085 (0.989−1.191)	0.085		-	-
	Continent	Europe	North America	297	0.136 (0.031−0.590)	0.008		0.150 (0.034−0.652)	0.011
	Continent	Europe	Asia		0.427 (0.156−1.167)	0.097		0.657 (0.221−1.947)	0.448

Infections were associated with higher reporting in the STR + VCR + ANTR + AMT regimen (ROR: 1.883, 95% CI: 1.095-3.237, p = 0.022; aROR: 1.967, 95% CI: 1.028-3.762, p = 0.041). Nervous system disorders showed higher reporting in the ASP+ group in the STR + VCR + AMT regimen (ROR: 3.818, 95% CI: 1.588-9.181, p = 0.003; aROR: 4.464, 95% CI: 1.615-12.342, p = 0.004) and in the STR + VCR + ANTR + AMT regimen (ROR: 2.653, 95% CI: 1.442-4.881, p = 0.002; aROR: 2.616, 95% CI: 1.397-4.898, p = 0.003). Pancreatitis also showed higher reporting with ASP addition in the STR + VCR + ANTR + AMT regimen (ROR: 4.886, 95% CI: 1.767-13.511, p = 0.002; aROR: 6.576, 95% CI: 2.208-19.590, p = 0.001). Gastrointestinal disorders showed higher reporting in the STR + VCR + ANTR + AMT regimen (ROR: 2.235, 95% CI: 1.340-3.729, p = 0.002; aROR: 2.544, 95% CI: 1.402-4.616, p = 0.002) and lower reporting in the STR + VCR + ANTR regimen (ROR: 0.370, 95% CI: 0.142-0.967, p = 0.043; aROR: 0.373, 95% CI: 0.141-0.990, p = 0.048). Hematologic disorders showed lower reporting in the ASP-containing STR + VCR + ANTR + ALKY + AMT regimen (ROR: 0.358, 95% CI: 0.160-0.802, p = 0.013; aROR: 0.393, 95% CI: 0.168-0.922, p = 0.032). Hepatobiliary disorders also showed lower reporting in the ASP-containing STR + VCR + ANTR + AMT regimen (ROR: 0.230, 95% CI: 0.092-0.572, p = 0.002; aROR: 0.230, 95% CI: 0.092-0.572, p = 0.002). Thrombotic disorders likewise showed lower reporting in the ASP-containing STR + VCR + ANTR + AMT regimen (ROR: 0.385, 95% CI: 0.174-0.850, p = 0.018; aROR: 0.383, 95% CI: 0.149-0.982, p = 0.046).

Sensitivity analyses

Sensitivity analyses reported the summary frequency for all AE categories, as well as the AE categories for which both ROR and aROR showed a statistically significant association. Continent-stratified analyses (Appendices F and G) showed broadly consistent regimen-specific patterns across Europe, North America, and Asia, although the precision of estimates was reduced in smaller subgroups. Exploratory analyses that included low-count regimen pairs and regimen pairs with concomitant prohibited drugs (Appendices H and I) were materially consistent with the primary analysis: the direction of association for the principal signals remained unchanged, with higher reporting of infections, nervous system disorders, pancreatitis, and gastrointestinal disorders in selected ASP-containing regimens and lower reporting of hepatobiliary and hematologic disorders in other regimen pairs. Extending the age definition to 0-17 years (Appendices J and K) altered statistical significance for some regimen-AE associations compared to the primary 0-14-year analysis. For example, STR + VCR + ANTR was associated with higher thrombotic disorder reporting in the 0-17-year analysis, whereas several associations significant in the 0-14-year analysis lost significance after age extension, including infections with STR + VCR + ANTR + ALKY + AMT. Overall, these sensitivity analyses confirmed the main findings across multiple scenarios while demonstrating variability according to population definition.

## Discussion

In this FAERS-based study, ASP-containing induction regimens in pediatric ALL showed regimen-specific AE reporting patterns rather than a uniform reporting pattern. Our findings extend prior cohort-based reports of ASP toxicity by showing that reporting patterns may differ according to the accompanying induction backbone, rather than being uniform across regimens. Our main finding was that ASP addition was associated with higher reporting of infections and nervous system disorders in several induction regimens, whereas hepatobiliary, gastrointestinal, hematologic, and thrombotic disorders were less frequently reported in selected comparisons. These findings suggest that the reporting pattern for ASP may depend on the accompanying drugs and the underlying regimen structure. This study does not replace cohort studies but complements them by generating regimen-specific pharmacovigilance hypotheses.

The increased reporting of infections in ASP-containing regimens is biologically plausible, given the known association of ASP with hypersensitivity and infection-related complications [8-11,20]. Although these findings are biologically plausible, they may also reflect notoriety bias in spontaneous reporting and a Weber effect-like pattern of selective reporting, whereby a well-known toxic drug is more likely to be implicated when adverse events occur in an ASP-containing regimen [21,22]. ASP can also alter the toxicity profile of combination chemotherapy by modifying protein synthesis and pharmacokinetics, which may contribute to infection-related reporting signals [8,23]. Similarly, the higher reporting of nervous system disorders in several ASP-containing regimens may reflect reporting bias as well as a hypothesis-generating signal, and should not be interpreted as evidence of a causal ASP effect [5,24-26]. The stronger signals observed in some regimen pairs, such as STR + VCR + AMT and STR + VCR + ANTR + AMT, should be interpreted cautiously as hypothesis-generating findings rather than evidence of a causal toxicity interaction. In particular, the STR + VCR + ANTR + AMT comparison showed a marked sex imbalance between the ASP-free and ASP-containing groups, and this imbalance may have contributed to the adjusted estimate despite multivariable adjustment. Nevertheless, the subgroup sizes were relatively small, and residual confounding cannot be completely excluded.

By contrast, hepatobiliary disorders were reported less frequently in some ASP-containing regimens, particularly STR + VCR + AMT and STR + VCR + ALKY + AMT. This finding should be interpreted cautiously, because the spontaneous reporting structure of FAERS does not allow reliable estimation of incidence. ASP-associated hepatotoxicity is a well-recognized AE, and previous studies in both adult and pediatric ALL have reported clinically relevant hepatotoxicity, pancreatitis, and thrombotic events in patients receiving ASP-containing regimens [27,28]. Accordingly, the lower reporting of hepatobiliary disorders in our study should not be interpreted as evidence of a protective effect of ASP. Rather, the most likely explanation is channeling bias and depletion-of-susceptibles: when hepatotoxicity is observed during ASP treatment, clinicians often discontinue or modify ASP, which may shift subsequent reports toward the ASP-free category and preferentially remove susceptible patients from the ASP+ group. Therefore, the lower reporting of hepatobiliary disorders should not be interpreted as a protective effect of ASP [27,28]. Gastrointestinal disorders were also less frequently reported in the ASP-containing STR + VCR + ANTR regimen, suggesting that the effect of ASP on gastrointestinal toxicity may not be uniform across induction base regimens. This interpretation is consistent with prior work showing that AE risks differ across pediatric ALL induction regimens and are influenced by the specific chemotherapy backbone [29]. In contrast, hematologic disorders showed both increased and decreased reporting depending on the regimen pair, underscoring the complexity of toxicity interactions among induction agents and suggesting that the net reporting pattern of ASP may depend on the surrounding treatment context [29]. Accordingly, the apparent associations observed in the adjusted analyses may partly reflect unmeasured differences in body size and dosing rather than regimen effects alone. Overall, the direction of association varied across AE categories, indicating that the observed signals were not uniform across outcomes and should be interpreted as regimen-specific reporting patterns rather than a single consistent ASP effect. The changes observed in the 0-17-year sensitivity analyses may partly reflect greater clinical heterogeneity among older adolescents, who may be managed under pediatric, adult, or pediatric-inspired protocols depending on local practice [15,16].

This study has several strengths. First, it used a large, multinational pharmacovigilance database spanning more than two decades, enabling the capture of rare safety signals in pediatric ALL. Second, the analysis focused on induction therapy and carefully excluded reports involving consolidation, maintenance, targeted therapy, or immunotherapy, which improved the specificity of the comparison. Third, we evaluated paired regimens that differed only by ASP inclusion, allowing a more clinically meaningful assessment of ASP-associated safety patterns than a broad unstratified comparison. Finally, the combination of unadjusted and adjusted analyses helped mitigate some of the bias introduced by demographic and regional differences in FAERS reporting.

This study also has several limitations. FAERS is a spontaneous reporting system and is subject to underreporting, reporting bias, duplicate submissions, and incomplete information. Because FAERS lacks a reliable exposure denominator, incidence cannot be estimated, and causal inference is limited. Although we restricted the analysis to induction-phase regimens and paired comparisons, important residual confounding may remain because detailed information on disease risk, treatment protocols, dosing, sequence of drug administration, clinical severity, body weight, and regional prescribing practices was unavailable. In particular, because weight data were missing for most reports, residual confounding related to body size and weight-dependent dosing cannot be excluded; therefore, the adjusted estimates should be interpreted cautiously. Because complete-case analysis preferentially retains reports with more complete covariate information, the adjusted analyses may reflect a selected subset of FAERS reports rather than the full pediatric ALL reporting population, which may limit the stability and generalizability of the adjusted estimates. The marked geographic imbalance between ASP+ and ASP- reports further suggests that continent-level adjustment is unlikely to fully account for differences in treatment protocols and reporting behavior across regions. Because ASP has a well-publicized toxicity profile, clinicians and reporters may disproportionately attribute AEs to ASP when it is present [21,22], which can inflate ASP+ signals across multiple outcome categories. In addition, notoriety bias and stimulated reporting may have influenced the magnitude of ASP-related reporting signals in FAERS, particularly because ASP is a well-recognized toxic agent in pediatric ALL. The most likely explanation for the lower reporting of hepatobiliary disorders is channeling bias and depletion-of-susceptibles [27,28]: patients who develop hepatotoxicity during ASP treatment are often switched to or continued on ASP-free regimens, which can preferentially deplete susceptible patients from the ASP+ group and shift subsequent reports toward the ASP-free category. Moreover, in the STR + VCR + ANTR + AMT comparison, the ASP-free group showed an unexplained female predominance, so residual sex confounding may have contributed to the observed nervous system aROR despite multivariable adjustment. Some estimates remained imprecise because several regimen-AE category combinations involved small cell counts, leading to wide confidence intervals. Although the models were intentionally parsimonious and used only a small number of clinically plausible covariates, the relatively small subgroup sizes may still have contributed to estimate instability. To guard against overinterpreting results driven by small-sample variability, we emphasized AE categories for which both unadjusted ROR and adjusted aROR showed concordant statistically significant associations. Therefore, these findings should be interpreted cautiously as exploratory reporting signals rather than definitive associations. Furthermore, this exploratory pharmacovigilance analysis evaluated multiple regimen-AE category combinations without applying a formal correction for multiple testing; therefore, the findings should be interpreted as hypothesis-generating signals rather than definitive comparative safety evidence. Accordingly, nominally significant associations, especially those based on small cell counts and wide confidence intervals, should be interpreted as preliminary reporting signals rather than definitive findings.

## Conclusions

In this FAERS-based pharmacovigilance study, ASP-containing induction regimens for pediatric ALL showed regimen-specific AE reporting signals rather than a single consistent pattern. Infections and nervous system disorders showed notable reporting signals in several ASP-containing regimens, whereas hepatobiliary, gastrointestinal, and thrombotic disorders were less frequently reported in selected comparisons. These findings are hypothesis-generating and should be interpreted as signal-detection results rather than evidence of causality, comparative safety, or incidence. Prospective studies are needed to confirm these observations and to clarify the clinical relevance of ASP across distinct induction regimens.

## Appendices

Appendix A

The ALL cohort was defined using the following preferred indication terms from the FAERS database, exactly as they are registered in MedDRA. Spelling was not modified to maintain consistency with the source data: acute lymphocytic leukaemia, acute lymphocytic leukaemia recurrent, Philadelphia positive acute lymphocytic leukaemia, acute lymphocytic leukaemia refractory, and acute lymphocytic leukaemia (in remission).

Appendix B

**Table 4:** Generic and brand names. All generic and brand names are listed exactly as registered in the United States Food and Drug Administration Adverse Event Reporting System (FAERS) database. Spelling was not modified to ensure consistency with the original terminology.

Study drug status	Class	Generic name	Brand name
Included drug	Asparaginase (ASP)	Asparaginase	Elspar, Erwinase, Erwinaze, Oncaspar, Spectrila
	Steroid (STR)	Prednisone, Prednisolone, Methylprednisolone	Aprednislon, Cortancyl, Decortin, Depo-Medrol, Medrol, Metilprednisolona, Prednisolon, Prednison, Prednisona, Predonine, Solu-Medrol, Solupred, Urbason
		Dexamethasone	Decadron, Dexametasona
		Hydrocortisone sodium phosphate	Hydrocortisone, Solu-Cortef
	Vinca alkaloid (VCR)	Vincristine	Oncovin
	Anthracycline (ANTR)	Daunorubicin	Cerubidine, Daunomycin
		Doxorubicin	Adriamycin, Adriblastina, Doxil, Doxorubicina
		Idarubicin	Idamycin
		Pirarubicin	Pinorubicin, Therarubicin
	Alkylating agent (ALKY)	Cyclophosphamide	Cytoxan, Endoxan, Genoxal, Procytox
	Antimetabolite (AMT)	Methotrexate	Ebetrexat, Rheumatrex, Trexall, Otrexup
Excluded drug	Pyrimidine analog (PYR)	Cytarabine	Cylocide
	Topoisomerase inhibitor (TOPO)	Etoposide	VePesi, Etopophos
	Purine analog (PNA)	Mercaptopurine	Purinethol
	Tyrosine kinase inhibitor (TKI)	Imatinib	Gleevec, Glivec
		Dasatinib	Sprycel
	Bispecific T‑cell engager antibody (BiTE)	Blinatumomab	Blincyto
	Anti‑CD20 antibody (CD20)	Rituximab	Mabthera, Rituxan

Appendix C

**Table 5:** Categorization of preferred terms. Preferred terms from the United States Food and Drug Administration Adverse Event Reporting System (FAERS) database are presented exactly as registered in the Medical Dictionary for Regulatory Activities. Spelling and capitalization were not modified to ensure consistency with the source data.

Adverse event category	Preferred terms
Infections	Abscess fungal, Adenovirus infection, Aspergillus infection, Bacterial infection, Bacterial sepsis, Bacteraemia, Brain abscess, Bronchopulmonary aspergillosis, Candida infection, Candida sepsis, Candidiasis, Cellulitis, Cerebral aspergillosis, Clostridium difficile infection, Congenital herpes simplex infection, COVID-19, Cytomegalovirus infection, Device related infection, Enterococcal infection, Escherichia infection, Escherichia sepsis, Escherichia urinary tract infection, Fungal infection, Fusarium infection, Herpes simplex, Herpes zoster, Mucormycosis, Oesophageal candidiasis, Peritonitis, Pneumonia, Pseudomonal sepsis, Pseudomonas infection, Respiratory tract infection, Sepsis, Septic shock, Staphylococcal bacteraemia, Staphylococcal infection, Systemic candida, Systemic inflammatory response syndrome, Urosepsis, Urinary tract infection, Varicella zoster virus infection
Nervous system disorders	Aphasia, Brain oedema, Brain injury, Cerebral atrophy, Confusional state, Delirium, Dizziness, Dysarthria, Electroencephalogram abnormal, Encephalopathy, Epilepsy, Eyelid ptosis, Facial paralysis, Facial paresis, Gait disturbance, Grand mal convulsion, Hallucination, Headache, Hemiparesis, Hypotonia, Incoherent, Lethargy, Leukoencephalopathy, Lennox-Gastaut syndrome, Loss of consciousness, Mental impairment, Mental status changes, Motor dysfunction, Muscular weakness, Neuralgia, Neurotoxicity, Neuropathy peripheral, Optic nerve disorder, Posterior reversible leukoencephalopathy syndrome, Reversible posterior leukoencephalopathy syndrome, Seizure, Somnolence, Status epilepticus, Tremor, Visual impairment
Hematologic disorders	Anaemia, Aplastic anaemia, Bone marrow failure, Cytopenia, Febrile bone marrow aplasia, Febrile neutropenia, Haematotoxicity, Haemoglobin decreased, Leukopenia, Myelosuppression, Neutropenia, Neutrophil count decreased, Pancytopenia, White blood cell count decreased
Hepatobiliary disorders	Adenoviral hepatitis, Alanine aminotransferase increased, Aspartate aminotransferase increased, Ascites, Blood bilirubin increased, Cholestasis, Gamma-glutamyltransferase increased, Hepatic encephalopathy, Hepatic failure, Hepatic steatosis, Hepatitis, Hepatocellular injury, Hepatomegaly, Hepatosplenomegaly, Hepatotoxicity, Hyperbilirubinaemia, Hypertransaminasaemia, Liver tenderness, Non-alcoholic steatohepatitis, Transaminases increased, Venoocclusive liver disease
Pancreatitis	Lipase increased, Pancreatic necrosis, Pancreatitis, Pancreatitis acute
Gastrointestinal disorders	Abdominal distension, Abdominal pain, Abdominal pain upper, Appendicitis, Appendicitis perforated, Caecitis, Colitis, Constipation, Decreased appetite, Diarrhoea, Dysphagia, Gastroenteritis viral, Gastrointestinal disorder, Gastrointestinal haemorrhage, Ileus, Ileus paralytic, Intussusception, Mucosal inflammation, Nausea, Oesophageal stenosis, Oral intake reduced, Stomatitis, Vomiting
Thrombotic disorders	Antithrombin III decreased, Cerebral infarction, Cerebral ischaemia, Cerebral thrombosis, Cerebral venous thrombosis, Cerebrovascular disorder, Coagulopathy, Disseminated intravascular coagulation, Haemorrhage intracranial, Hypofibrinogenaemia, Intracranial venous sinus thrombosis, Ischaemic stroke, Pulmonary embolism
Metabolic and endocrine disorders	Ammonia increased, Blood cholesterol increased, Blood pressure increased, Dehydration, Diabetes mellitus, Diabetic ketoacidosis, Electrolyte imbalance, Hyperglycaemia, Hyperlipidaemia, Hypertriglyceridaemia, Hypocalcaemia, Hypoglycaemia, Hypokalaemia, Hyponatraemia, Inappropriate antidiuretic hormone secretion, Weight decreased, Weight increased
Hypersensitivity	Drug hypersensitivity, Hypersensitivity
Respiratory disorders	Acute respiratory distress syndrome, Dyspnoea, Hypoxia, Pleural effusion, Pneumonitis, Pneumothorax, Pulmonary haemorrhage, Respiratory distress, Respiratory failure, Sinusitis

Appendix D

**Table 6:** MedDRA versions used for AERS and FAERS datasets by time period. AERS, Adverse Event Reporting System; FAERS, United States Food and Drug Administration Adverse Event Reporting System; MedDRA, Medical Dictionary for Regulatory Activities.

Data	Time period	MedDRA version
AERS	2004Q1−2012Q3	No information on the MedDRA version used
FAERS	2012Q4	16.0
	2013Q1–2013Q2	16.1
	2013Q3–2014Q2	17.0
	2014Q3	17.1
	2014Q4–2015Q2	18.0
	2015Q3–2015Q4	18.1
	2016Q1–2016Q2	19.0
	2016Q3–2017Q1	19.1
	2017Q2	20.0
	2017Q3–2017Q4	20.1
	2018Q1–2018Q3	21.0
	2018Q4–2019Q1	21.1
	2019Q2–2019Q3	22.0
	2019Q4–2020Q1	22.1
	2020Q2	23.0
	2020Q3–2021Q1	23.1
	2021Q2–2021Q3	24.0
	2021Q4–2022Q1	24.1
	2022Q2–2022Q3	25.0
	2022Q4–2023Q1	25.1
	2023Q2–2023Q3	26.0
	2023Q4–2024Q1	26.1
	2024Q2–2024Q3	27.0
	2024Q4–2025Q1	27.1
	2025Q2–2025Q3	28.0
	2025Q4	28.1

Appendix E

**Table 7:** Regimen pairs excluded from the primary analysis because of insufficient case counts or concomitant prohibited drugs. The numbers indicate the number of cases for each regimen group. ALKY, alkylating agent; AMT, antimetabolite; ANTR, anthracycline; ASP-, asparaginase not added to base regimen; ASP+, asparaginase added to base regimen; BiTE, bispecific T-cell engager antibody; PNA, pyrimidine analog; STR, steroid; TKI, tyrosine kinase inhibitor; TOPO, topoisomerase inhibitor; VCR, vincristine.

Base regimen	ASP-	ASP+
Regimen pairs with 10 to 39 cases in at least one group		
STR + VCR	21	30
STR + VCR + ALKY + AMT	17	11
Regimen pairs with prohibited drugs and at least 10 cases in both groups		
STR + VCR + AMT + PNA	237	24
STR + VCR + ANTR + ALKY + AMT + PNA	37	64
STR + VCR + ANTR + ALKY + AMT + TOPO + PNA	10	71
STR + VCR + ANTR + AMT + PNA	24	21

Appendix F

**Table 8:** Reporting frequencies of adverse event categories by induction regimen, asparaginase use, and continent. Data are summarized as frequencies (reporting proportions). Cases could contribute to more than one adverse event category if events from multiple categories were reported. Within each category, each case was counted only once, regardless of the number of preferred terms reported. ALKY, alkylating agent; AMT, antimetabolite; ANTR, anthracycline; ASP-, asparaginase not added to base regimen; ASP+, asparaginase added to base regimen; STR, steroid; VCR, vincristine.

Base regimen	STR + VCR + ANTR		STR + VCR + AMT		STR + VCR + ANTR + AMT		STR + VCR + ANTR + ALKY + AMT	
	ASP-	ASP+	ASP-	ASP+	ASP-	ASP+	ASP-	ASP+
Europe	N = 41	N = 33	N = 63	N = 8	N = 113	N = 44	N = 25	N = 10
Infections	7 (17.1)	14 (42.4)	10 (15.9)	0 (0.0)	13 (11.5)	12 (27.3)	0 (0.0)	1 (10.0)
Nervous system disorders	2 (4.9)	7 (21.2)	4 (6.3)	5 (62.5)	5 (4.4)	12 (27.3)	5 (20.0)	3 (30.0)
Hematologic disorders	19 (46.3)	11 (33.3)	12 (19.0)	0 (0.0)	23 (20.4)	18 (40.9)	10 (40.0)	3 (30.0)
Hepatobiliary disorders	5 (12.2)	9 (27.3)	19 (30.2)	0 (0.0)	25 (22.1)	1 (2.3)	5 (20.0)	2 (20.0)
Pancreatitis	3 (7.3)	0 (0.0)	0 (0.0)	0 (0.0)	3 (2.7)	3 (6.8)	4 (16.0)	0 (0.0)
Gastrointestinal disorders	9 (22.0)	5 (15.2)	7 (11.1)	0 (0.0)	22 (19.5)	21 (47.7)	3 (12.0)	2 (20.0)
Thrombotic disorders	0 (0.0)	5 (15.2)	19 (30.2)	1 (12.5)	21 (18.6)	5 (11.4)	5 (20.0)	1 (10.0)
Metabolic and endocrine disorders	5 (12.2)	1 (3.0)	4 (6.3)	0 (0.0)	11 (9.7)	10 (22.7)	5 (20.0)	1 (10.0)
Hypersensitivity	0 (0.0)	1 (3.0)	0 (0.0)	0 (0.0)	1 (0.9)	1 (2.3)	0 (0.0)	0 (0.0)
Respiratory disorders	0 (0.0)	0 (0.0)	1 (1.6)	0 (0.0)	3 (2.7)	0 (0.0)	2 (8.0)	0 (0.0)
North America	N = 5	N = 8	N = 33	N = 15	N = 44	N = 32	N = 14	N = 5
Infections	0 (0.0)	2 (25.0)	12 (36.4)	4 (26.7)	10 (22.7)	9 (28.1)	2 (14.3)	1 (20.0)
Nervous system disorders	0 (0.0)	1 (12.5)	5 (15.2)	5 (33.3)	9 (20.5)	5 (15.6)	0 (0.0)	0 (0.0)
Hematologic disorders	1 (20.0)	4 (50.0)	4 (12.1)	6 (40.0)	14 (31.8)	8 (25.0)	9 (64.3)	3 (60.0)
Hepatobiliary disorders	0 (0.0)	0 (0.0)	0 (0.0)	2 (13.3)	3 (6.8)	2 (6.2)	0 (0.0)	0 (0.0)
Pancreatitis	0 (0.0)	0 (0.0)	5 (15.2)	0 (0.0)	2 (4.5)	11 (34.4)	0 (0.0)	0 (0.0)
Gastrointestinal disorders	1 (20.0)	0 (0.0)	1 (3.0)	2 (13.3)	11 (25.0)	5 (15.6)	1 (7.1)	1 (20.0)
Thrombotic disorders	1 (20.0)	0 (0.0)	0 (0.0)	3 (20.0)	2 (4.5)	0 (0.0)	0 (0.0)	0 (0.0)
Metabolic and endocrine disorders	0 (0.0)	0 (0.0)	2 (6.1)	1 (6.7)	11 (25.0)	8 (25.0)	1 (7.1)	0 (0.0)
Hypersensitivity	0 (0.0)	0 (0.0)	0 (0.0)	0 (0.0)	1 (2.3)	0 (0.0)	0 (0.0)	0 (0.0)
Respiratory disorders	0 (0.0)	0 (0.0)	3 (9.1)	1 (6.7)	1 (2.3)	2 (6.2)	0 (0.0)	0 (0.0)
Asia	N = 1	N = 48	N = 17	N = 17	N = 16	N = 48	N = 3	N = 45
Infections	0 (0.0)	7 (14.6)	3 (17.6)	1 (5.9)	6 (37.5)	12 (25.0)	2 (66.7)	11 (24.4)
Nervous system disorders	0 (0.0)	11 (22.9)	2 (11.8)	4 (23.5)	5 (31.2)	13 (27.1)	0 (0.0)	17 (37.8)
Hematologic disorders	0 (0.0)	4 (8.3)	10 (58.8)	4 (23.5)	9 (56.2)	16 (33.3)	2 (66.7)	4 (8.9)
Hepatobiliary disorders	1 (100.0)	2 (4.2)	3 (17.6)	0 (0.0)	0 (0.0)	2 (4.2)	0 (0.0)	3 (6.7)
Pancreatitis	0 (0.0)	3 (6.2)	0 (0.0)	0 (0.0)	0 (0.0)	2 (4.2)	0 (0.0)	1 (2.2)
Gastrointestinal disorders	0 (0.0)	3 (6.2)	3 (17.6)	0 (0.0)	0 (0.0)	16 (33.3)	0 (0.0)	3 (6.7)
Thrombotic disorders	0 (0.0)	9 (18.8)	1 (5.9)	1 (5.9)	3 (18.8)	2 (4.2)	0 (0.0)	11 (24.4)
Metabolic and endocrine disorders	0 (0.0)	7 (14.6)	2 (11.8)	4 (23.5)	0 (0.0)	7 (14.6)	0 (0.0)	7 (15.6)
Hypersensitivity	0 (0.0)	0 (0.0)	0 (0.0)	0 (0.0)	0 (0.0)	0 (0.0)	0 (0.0)	0 (0.0)
Respiratory disorders	0 (0.0)	2 (4.2)	0 (0.0)	1 (5.9)	3 (18.8)	2 (4.2)	0 (0.0)	2 (4.4)

Appendix G

**Table 9:** The ROR and aROR of adverse event categories by continent. N indicates the number of reports included in each model. Only adverse event categories with both ROR and aROR showing statistically significant associations were included. Multivariate models were selected based on the lowest Akaike information criterion among candidate models. ALKY, alkylating agent; AMT, antimetabolite; ANTR, anthracycline; aROR, adjusted reporting odds ratio; ASP+, asparaginase added to base regimen; CI, confidence interval; ROR, reporting odds ratio; STR, steroid; VCR, vincristine; -, not selected in multivariate analysis.

Continent	Adverse event category	Variable	Reference	Comparison	Univariate analysis			Multivariate analysis		
					N	ROR (95% CI)	p-value	N	aROR (95% CI)	p-value
Europe	Infections	Regimen	STR + VCR + ANTR	ASP+	74	3.579 (1.231−10.402)	0.019	72	3.754 (1.248−11.286)	0.019
		Sex	Female	Male	72	3.224 (0.950−10.943)	0.060		2.943 (0.831−10.428)	0.094
		Age	Per 1-year increase		74	0.888 (0.767−1.028)	0.112		-	-
		Regimen	STR + VCR + ANTR + AMT	ASP+	157	2.885 (1.197−6.953)	0.018	157	2.848 (1.170−6.937)	0.021
		Sex	Female	Male	157	1.889 (0.779−4.577)	0.159		-	-
		Age	Per 1-year increase		157	0.897 (0.795−1.012)	0.077		0.897 (0.791−1.017)	0.089
	Nervous system disorders	Regimen	STR + VCR + ANTR	ASP+	74	5.250 (1.010−27.279)	0.049	74	5.250 (1.010−27.279)	0.049
		Sex	Female	Male	72	0.191 (0.034−1.066)	0.059		-	-
		Age	Per 1-year increase		74	0.882 (0.710−1.096)	0.257		-	-
		Regimen	STR + VCR + AMT	ASP+	71	24.583 (4.258−141.942)	<0.001	71	24.583 (4.258−141.942)	<0.001
		Sex	Female	Male	70	0.652 (0.145−2.933)	0.577		-	-
		Age	Per 1-year increase		71	1.152 (0.948−1.399)	0.155		-	-
		Regimen	STR + VCR + ANTR + AMT	ASP+	157	8.100 (2.655−24.708)	<0.001	157	8.437 (2.735−26.024)	<0.001
		Sex	Female	Male	157	0.840 (0.306−2.301)	0.734		-	-
		Age	Per 1-year increase		157	1.034 (0.903−1.185)	0.630		1.062 (0.912−1.237)	0.439
	Hematologic disorders	Regimen	STR + VCR + ANTR + AMT	ASP+	157	2.709 (1.273−5.767)	0.010	157	2.709 (1.273−5.767)	0.010
		Sex	Female	Male	157	0.596 (0.290−1.225)	0.159		-	-
		Age	Per 1-year increase		157	0.922 (0.835−1.017)	0.105		-	-
	Hepatobiliary disorders	Regimen	STR + VCR + ANTR + AMT	ASP+	157	0.082 (0.011−0.624)	0.016	157	0.082 (0.011−0.624)	0.016
		Sex	Female	Male	157	0.447 (0.186−1.076)	0.073		-	-
		Age	Per 1-year increase		157	1.035 (0.924−1.160)	0.549		-	-
	Gastrointestinal disorders	Regimen	STR + VCR + ANTR + AMT	ASP+	157	3.777 (1.779−8.018)	0.001	157	3.750 (1.753−8.021)	0.001
		Sex	Female	Male	157	0.890 (0.441−1.795)	0.744		-	-
		Age	Per 1-year increase		157	0.919 (0.834−1.013)	0.088		0.919 (0.829−1.019)	0.108
	Metabolic and endocrine disorders	Regimen	STR + VCR + ANTR + AMT	ASP+	157	2.727 (1.065−6.983)	0.036	157	2.727 (1.065−6.983)	0.036
		Sex	Female	Male	157	1.674 (0.652−4.296)	0.284		-	-
		Age	Per 1-year increase		157	1.090 (0.960−1.236)	0.183		-	-
North America	Hematologic disorders	Regimen	STR + VCR + AMT	ASP+	48	4.833 (1.112−21.014)	0.036	48	4.833 (1.112−21.014)	0.036
		Sex	Female	Male	38	0.711 (0.160−3.163)	0.654		-	-
		Age	Per 1-year increase		48	1.026 (0.842−1.251)	0.797		-	-
	Pancreatitis	Regimen	STR + VCR + ANTR + AMT	ASP+	76	11.000 (2.232−54.210)	0.003	76	11.000 (2.232−54.210)	0.003
		Sex	Female	Male	76	0.431 (0.120−1.544)	0.196		-	-
		Age	Per 1-year increase		76	1.101 (0.949−1.278)	0.206		-	-
Asia	Hematologic disorders	Regimen	STR + VCR + ANTR + ALKY + AMT	ASP+	48	0.049 (0.004−0.664)	0.023	48	0.049 (0.004−0.664)	0.023
		Sex	Female	Male	48	1.500 (0.247−9.110)	0.660		-	-
		Age	Per 1-year increase		48	1.405 (1.032−1.913)	0.031		-	-

Appendix H

**Table 10:** Reporting frequencies of adverse event categories by induction regimen and asparaginase use in extended analyses, including low-count and prohibited-drug regimen pairs. Data are summarized as frequencies (reporting proportions). Cases could contribute to more than one adverse event category if events from multiple categories were reported. Within each category, each case was counted only once, regardless of the number of preferred terms reported. ALKY, alkylating agent; AMT, antimetabolite; ANTR, anthracycline; ASP-, asparaginase not added to base regimen; ASP+, asparaginase added to base regimen; BiTE, bispecific T-cell engager antibody; PNA, pyrimidine analog; STR, steroid; TKI, tyrosine kinase inhibitor; TOPO, topoisomerase inhibitor; VCR, vincristine.

Base regimen	STR + VCR		STR + VCR + ALKY + AMT		STR + VCR + AMT + PNA		STR + VCR + ANTR + ALKY + AMT + PNA		STR + VCR + ANTR + ALKY + AMT + TOPO + PNA		STR + VCR + ANTR + AMT + PNA	
	ASP-	ASP+	ASP-	ASP+	ASP-	ASP+	ASP-	ASP+	ASP-	ASP+	ASP-	ASP+
	N = 21	N = 30	N = 17	N = 11	N = 237	N = 24	N = 37	N = 64	N = 10	N = 71	N = 24	N = 21
Infections	4 (19.0)	16 (53.3)	5 (29.4)	2 (18.2)	28 (11.8)	3 (12.5)	9 (24.3)	9 (14.1)	5 (50.0)	49 (69.0)	1 (4.2)	1 (4.8)
Nervous system disorders	3 (14.3)	5 (16.7)	4 (23.5)	5 (45.5)	16 (6.8)	3 (12.5)	4 (10.8)	14 (21.9)	0 (0.0)	0 (0.0)	3 (12.5)	8 (38.1)
Hematologic disorders	1 (4.8)	6 (20.0)	8 (47.1)	3 (27.3)	80 (33.8)	8 (33.3)	16 (43.2)	9 (14.1)	2 (20.0)	16 (22.5)	13 (54.2)	1 (4.8)
Hepatobiliary disorders	2 (9.5)	1 (3.3)	5 (29.4)	2 (18.2)	46 (19.4)	1 (4.2)	5 (13.5)	4 (6.2)	0 (0.0)	1 (1.4)	1 (4.2)	2 (9.5)
Pancreatitis	1 (4.8)	2 (6.7)	0 (0.0)	0 (0.0)	5 (2.1)	6 (25.0)	0 (0.0)	1 (1.6)	0 (0.0)	0 (0.0)	4 (16.7)	0 (0.0)
Gastrointestinal disorders	2 (9.5)	2 (6.7)	2 (11.8)	4 (36.4)	20 (8.4)	6 (25.0)	5 (13.5)	4 (6.2)	0 (0.0)	1 (1.4)	1 (4.2)	2 (9.5)
Thrombotic disorders	2 (9.5)	4 (13.3)	0 (0.0)	2 (18.2)	5 (2.1)	4 (16.7)	2 (5.4)	0 (0.0)	0 (0.0)	0 (0.0)	0 (0.0)	1 (4.8)
Metabolic and endocrine disorders	3 (14.3)	2 (6.7)	2 (11.8)	3 (27.3)	23 (9.7)	1 (4.2)	3 (8.1)	4 (6.2)	5 (50.0)	2 (2.8)	4 (16.7)	0 (0.0)
Hypersensitivity	1 (4.8)	1 (3.3)	0 (0.0)	0 (0.0)	7 (3.0)	0 (0.0)	1 (2.7)	0 (0.0)	0 (0.0)	1 (1.4)	0 (0.0)	1 (4.8)
Respiratory disorders	2 (9.5)	6 (20.0)	1 (5.9)	1 (9.1)	7 (3.0)	1 (4.2)	3 (8.1)	3 (4.7)	0 (0.0)	1 (1.4)	1 (4.2)	0 (0.0)

Appendix I

**Table 11:** The ROR and aROR of adverse event categories in extended analyses including low-count and prohibited-drug regimen pairs N indicates the number of reports included in each model. Only adverse event categories with both ROR and aROR showing statistically significant associations were included. Multivariate models were selected based on the lowest Akaike information criterion among candidate models. †, not calculated due to zero cell count; -, not selected in multivariate analysis; ALKY, alkylating agent; AMT, antimetabolite; ANTR, anthracycline; aROR, adjusted reporting odds ratio; ASP+, asparaginase added to base regimen; CI, confidence interval; PNA, pyrimidine analog; ROR, reporting odds ratio; STR, steroid; TOPO, topoisomerase inhibitor; VCR, vincristine.

Adverse event category	Variable	Reference	Comparison	Univariate analysis			Multivariate analysis		
				N	ROR (95% CI)	p-value	N	aROR (95% CI)	p-value
Hematologic disorders	Regimen	STR + VCR + ANTR + ALKY + AMT + PNA	ASP+	101	0.215 (0.082−0.560)	0.002	82	0.211 (0.065−0.682)	0.009
	Sex	Female	Male	100	1.113 (0.450−2.753)	0.817		-	-
	Age	Per 1-year increase		101	0.845 (0.745−0.958)	0.008		0.833 (0.717−0.969)	0.018
	Continent	Europe	North America	82	3.378 (1.038−10.992)	0.043		3.160 (0.831−12.014)	0.091
	Continent	Europe	Asia		1.169 (0.312−4.375)	0.816		1.502 (0.348−6.475)	0.585
	Regimen	STR + VCR + ANTR + AMT + PNA	ASP+	45	0.042 (0.005−0.368)	0.004	41	0.037 (0.004−0.373)	0.005
	Sex	Female	Male	41	0.225 (0.051−1.000)	0.050		0.336 (0.056−2.022)	0.234
	Age	Per 1-year increase		45	0.861 (0.704−1.053)	0.144		0.826 (0.639−1.069)	0.146
	Continent	Europe	North America	42	0.088 (0.017−0.451)	0.004		-	-
	Continent	Europe	Asia		†	†		-	-
Pancreatitis	Regimen	STR + VCR + AMT + PNA	ASP+	261	15.467 (4.300−55.629)	<0.001	249	10.834 (2.221−52.860)	0.003
	Sex	Female	Male	249	1.820 (0.360−9.209)	0.469		2.391 (0.443−12.900)	0.311
	Age	Per 1-year increase		261	0.755 (0.563−1.013)	0.061		0.802 (0.590−1.091)	0.161
	Continent	Europe	North America	244	0.292 (0.075−1.133)	0.075		-	-
	Continent	Europe	Asia		†	†		-	-
Thrombotic disorders	Regimen	STR + VCR + AMT + PNA	ASP+	261	9.280 (2.307−37.326)	0.002	249	8.573 (1.859−39.536)	0.006
	Sex	Female	Male	249	0.993 (0.232−4.256)	0.993		1.153 (0.258−5.153)	0.852
	Age	Per 1-year increase		261	1.088 (0.887−1.334)	0.417		-	-
	Continent	Europe	North America	244	0.223 (0.045−1.100)	0.065		-	-
	Continent	Europe	Asia		†	†		-	-
Metabolic and endocrine disorders	Regimen	STR + VCR + ANTR + ALKY + AMT + TOPO + PNA	ASP+	81	0.029 (0.004−0.189)	<0.001	81	0.049 (0.005−0.509)	0.012
	Sex	Female	Male	39	3.654 (0.613−21.775)	0.155		-	-
	Age	Per 1-year increase		81	1.608 (1.153−2.243)	0.005		1.535 (1.079−2.185)	0.017
	Continent	Europe	North America		†	†		-	-
	Continent	Europe	Asia		†	†		-	-

Appendix J

**Table 12:** Reporting frequencies of adverse event categories by induction regimen and asparaginase use among reports aged 0-17 years. Data are summarized as frequencies (reporting proportions). Cases could contribute to more than one adverse event category if events from multiple categories were reported. Within each category, each case was counted only once, regardless of the number of preferred terms reported. ALKY, alkylating agent; AMT, antimetabolite; ANTR, anthracycline; ASP-, asparaginase not added to base regimen; ASP+, asparaginase added to base regimen; STR, steroid; VCR, vincristine.

Base regimen	STR + VCR + ANTR		STR + VCR + AMT		STR + VCR + ANTR + AMT		STR + VCR + ANTR + ALKY + AMT	
	ASP-	ASP+	ASP-	ASP+	ASP-	ASP+	ASP-	ASP+
	N = 65	N = 114	N = 123	N = 52	N = 202	N = 159	N = 50	N = 73
Infections	13 (20.0)	33 (28.9)	28 (22.8)	11 (21.2)	35 (17.3)	43 (27.0)	4 (8.0)	21 (28.8)
Nervous system disorders	5 (7.7)	23 (20.2)	12 (9.8)	16 (30.8)	22 (10.9)	37 (23.3)	7 (14.0)	21 (28.8)
Hematologic disorders	23 (35.4)	27 (23.7)	27 (22.0)	13 (25.0)	53 (26.2)	54 (34.0)	21 (42.0)	17 (23.3)
Hepatobiliary disorders	8 (12.3)	14 (12.3)	23 (18.7)	5 (9.6)	33 (16.3)	6 (3.8)	5 (10.0)	5 (6.8)
Pancreatitis	5 (7.7)	5 (4.4)	5 (4.1)	0 (0.0)	8 (4.0)	22 (13.8)	4 (8.0)	1 (1.4)
Gastrointestinal disorders	13 (20.0)	11 (9.6)	11 (8.9)	4 (7.7)	37 (18.3)	52 (32.7)	4 (8.0)	12 (16.4)
Thrombotic disorders	2 (3.1)	21 (18.4)	20 (16.3)	8 (15.4)	31 (15.3)	10 (6.3)	7 (14.0)	12 (16.4)
Metabolic and endocrine disorders	6 (9.2)	13 (11.4)	8 (6.5)	6 (11.5)	27 (13.4)	29 (18.2)	7 (14.0)	9 (12.3)
Hypersensitivity	0 (0.0)	1 (0.9)	0 (0.0)	0 (0.0)	4 (2.0)	1 (0.6)	0 (0.0)	0 (0.0)
Respiratory disorders	4 (6.2)	7 (6.1)	6 (4.9)	6 (11.5)	10 (5.0)	8 (5.0)	2 (4.0)	4 (5.5)

Appendix K

**Table 13:** The ROR and aROR of adverse event categories among reports aged 0-17 years. N indicates the number of reports included in each model. Only adverse event categories with both ROR and aROR showing statistically significant associations were included. Multivariate models were selected based on the lowest Akaike information criterion among candidate models. AMT, antimetabolite; ANTR, anthracycline; aROR, adjusted reporting odds ratio; ASP+, asparaginase added to base regimen; CI, confidence interval; ROR, reporting odds ratio; STR, steroid; VCR, vincristine; -, not selected in multivariate analysis.

Adverse event category	Variable	Reference	Comparison	Univariate analysis			Multivariate analysis		
				N	ROR (95% CI)	p-value	N	aROR (95% CI)	p-value
Nervous system disorders	Regimen	STR + VCR + AMT	ASP+	175	4.111 (1.779−9.499)	0.001	152	5.107 (1.930−13.511)	0.001
	Sex	Female	Male	163	0.747 (0.323−1.730)	0.496		0.966 (0.384−2.430)	0.941
	Age	Per 1-year increase		175	1.090 (0.991−1.199)	0.076		-	-
	Continent	Europe	North America	163	1.718 (0.682−4.326)	0.251		1.320 (0.468−3.720)	0.599
	Continent	Europe	Asia		1.350 (0.447−4.079)	0.595		0.627 (0.179−2.198)	0.466
Hepatobiliary disorders	Regimen	STR + VCR + ANTR + AMT	ASP+	361	0.201 (0.082−0.492)	<0.001	337	0.252 (0.093−0.685)	0.007
	Sex	Female	Male	354	0.864 (0.444−1.682)	0.667		-	-
	Age	Per 1-year increase		361	1.015 (0.944−1.091)	0.696		-	-
	Continent	Europe	North America	337	0.450 (0.189−1.072)	0.071		0.526 (0.218−1.271)	0.153
	Continent	Europe	Asia		0.155 (0.036−0.670)	0.013		0.279 (0.061−1.268)	0.099
Pancreatitis	Regimen	STR + VCR + ANTR + AMT	ASP+	361	3.894 (1.684−9.004)	0.001	337	5.265 (2.118−13.089)	<0.001
	Sex	Female	Male	354	0.949 (0.448−2.009)	0.892		-	-
	Age	Per 1-year increase		361	1.136 (1.041−1.239)	0.004		1.127 (1.024−1.240)	0.015
	Continent	Europe	North America	337	4.549 (1.937−10.687)	0.001		3.359 (1.368−8.248)	0.008
	Continent	Europe	Asia		0.567 (0.119−2.694)	0.476		0.268 (0.054−1.341)	0.109
Gastrointestinal disorders	Regimen	STR + VCR + ANTR + AMT	ASP+	361	2.167 (1.332−3.526)	0.002	330	2.476 (1.407−4.357)	0.002
	Sex	Female	Male	354	0.809 (0.500−1.308)	0.387		0.795 (0.474−1.332)	0.383
	Age	Per 1-year increase		361	0.955 (0.906−1.007)	0.092		-	-
	Continent	Europe	North America	337	0.779 (0.425−1.430)	0.421		0.654 (0.349−1.229)	0.187
	Continent	Europe	Asia		0.854 (0.446−1.637)	0.635		0.611 (0.294−1.269)	0.187
Thrombotic disorders	Regimen	STR + VCR + ANTR	ASP+	179	7.113 (1.611−31.411)	0.010	164	9.435 (1.790−49.746)	0.008
	Sex	Female	Male	174	3.060 (1.074−8.716)	0.036		4.054 (1.294−12.694)	0.016
	Age	Per 1-year increase		179	1.078 (0.994−1.171)	0.071		1.085 (0.986−1.194)	0.095
	Continent	Europe	North America	169	3.850 (1.121−13.223)	0.032		4.013 (0.895−17.997)	0.070
	Continent	Europe	Asia		2.881 (1.001−8.293)	0.050		1.721 (0.505−5.864)	0.386

## References

[1] Ding F, Deng L, Xiong J, Cheng Z, Xu J (2025). Analysis of global trends in acute lymphoblastic leukemia in children aged 0-5 years from 1990 to 2021. Front Pediatr.

[2] Hu Y, Liu Y, Fu J, Liu Y, Wang H, Song Y (2024). Global, regional, and national burden of acute lymphoblastic leukemia in children: epidemiological trends analysis from 1990 to 2021. iScience.

[3] Sebastian G (2024). How I treat newly diagnosed acute lymphoblastic leukemia. Clin Hematol Int.

[4] Tucci F, Aricò M (2008). Treatment of pediatric acute lymphoblastic leukemia. Haematologica.

[5] Ronghe M, Burke GA, Lowis SP, Estlin EJ (2001). Remission induction therapy for childhood acute lymphoblastic leukaemia: clinical and cellular pharmacology of vincristine, corticosteroids, L-asparaginase and anthracyclines. Cancer Treat Rev.

[6] Pui CH, Evans WE (2013). A 50-year journey to cure childhood acute lymphoblastic leukemia. Semin Hematol.

[7] Koprivnikar J, McCloskey J, Faderl S (2017). Safety, efficacy, and clinical utility of asparaginase in the treatment of adult patients with acute lymphoblastic leukemia. Onco Targets Ther.

[8] Hijiya N, van der Sluis IM (2016). Asparaginase-associated toxicity in children with acute lymphoblastic leukemia. Leuk Lymphoma.

[9] Schmiegelow K, Rank CU, Stock W, Dworkin E, van der Sluis I (2021). SOHO state of the art updates and next questions: management of asparaginase toxicity in adolescents and young adults with acute lymphoblastic leukemia. Clin Lymphoma Myeloma Leuk.

[10] Schmidt MP, Ivanov AV, Coriu D, Miron IC (2021). L-asparaginase toxicity in the treatment of children and adolescents with acute lymphoblastic leukemia. J Clin Med.

[11] Awwad S, Alnasr RA, Almanjomi F, Al Sultan M, Howaidi J, Almotairi M, AlFayyad I (2024). Peg-asparaginase associated toxicities in children with acute lymphoblastic leukemia: a single-center cross-sectional study. Pediatr Hematol Oncol J.

[12] (2026). FDA Adverse Event Monitoring System (AEMS) quarterly data extract files. https://fis.fda.gov/extensions/FPD-QDE-FAERS/FPD-QDE-FAERS.html.

[13] Benchimol EI, Smeeth L, Guttmann A (2015). The REporting of studies Conducted using Observational Routinely-collected health Data (RECORD) statement. PLoS Med.

[14] Public Policy Committee, International Society of Pharmacoepidemiology (2016). Guidelines for good pharmacoepidemiology practice (GPP). Pharmacoepidemiol Drug Saf.

[15] Ribera JM, Ribera J, Genescà E (2014). Treatment of adolescent and young adults with acute lymphoblastic leukemia. Mediterr J Hematol Infect Dis.

[16] Carobolante F, Chiaretti S, Skert C, Bassan R (2020). Practical guidance for the management of acute lymphoblastic leukemia in the adolescent and young adult population. Ther Adv Hematol.

[17] Ogura T, Shiraishi C (2025). Comparative analysis of adverse event profiles among seven statins for hypercholesterolemia management using the United States FDA Adverse Event Reporting System. Cureus.

[18] Ogura T, Shiraishi C (2025). Comparison of adverse events among angiotensin receptor blockers in hypertension using the United States Food and Drug Administration Adverse Event Reporting System. Cureus.

[19] Akaike H (2003). A new look at the statistical model identification. IEEE Trans Autom Control.

[20] Varela Gonzalez-Aller J, Nadal P, Cañizares S (2025). Asparaginase hypersensitivity reactions in NK/T-cell lymphomas. Clin Pract.

[21] Neha R, Subeesh V, Beulah E, Gouri N, Maheswari E (2021). Existence of notoriety bias in FDA Adverse Event Reporting System Database and its impact on signal strength. Hosp Pharm.

[22] Hartnell NR, Wilson JP (2004). Replication of the Weber effect using postmarketing adverse event reports voluntarily submitted to the United States Food and Drug Administration. Pharmacotherapy.

[23] Asselin B, Rizzari C (2015). Asparaginase pharmacokinetics and implications of therapeutic drug monitoring. Leuk Lymphoma.

[24] Heidari S, Panahishokouh M, Babakhani D (2023). Vincristine-induced seizure from drug interactions: a case report and review of literature. Case Rep Oncol.

[25] Dietrich J, Rao K, Pastorino S, Kesari S (2011). Corticosteroids in brain cancer patients: benefits and pitfalls. Expert Rev Clin Pharmacol.

[26] Anastasopoulou S, Swann G, Andres-Jensen L (2024). Severe steroid-related neuropsychiatric symptoms during paediatric acute lymphoblastic leukaemia therapy—an observational Ponte di Legno Toxicity Working Group study. Br J Haematol.

[27] Aldoss I, Douer D, Behrendt CE, Chaudhary P, Mohrbacher A, Vrona J, Pullarkat V (2016). Toxicity profile of repeated doses of PEG-asparaginase incorporated into a pediatric-type regimen for adult acute lymphoblastic leukemia. Eur J Haematol.

[28] Christ TN, Stock W, Knoebel RW (2018). Incidence of asparaginase-related hepatotoxicity, pancreatitis, and thrombotic events in adults with acute lymphoblastic leukemia treated with a pediatric-inspired regimen. J Oncol Pharm Pract.

[29] West ZE, Castellino SM, Monroe C, Thomas AS, McCracken C, Miller TP (2021). Quantifying the difference in risk of adverse events by induction treatment regimen in pediatric acute lymphoblastic leukemia. Leuk Lymphoma.

